# Barriers to Inpatient Psychiatry Research in Palestine

**DOI:** 10.1192/bjo.2026.11271

**Published:** 2026-06-30

**Authors:** Nizar Marzouqa, Samah AbuAjamia, Walaa Alnammourah, Iltizam Morrar

**Affiliations:** Bethlehem Psychiatric Hospital, Bethlehem, Palestine

## Abstract

**Aims:**

To explore the main barriers facing Palestinian mental health professionals conducting inpatient psychiatry research in Palestine.

**Methods:**

A qualitative approach, through unstructured interviews with medical professionals who worked on academic papers at Bethlehem Psychiatric Hospital (BPH).

**Results:**

Four themes of challenges to inpatient psychiatric research in Palestine were identified:

Theme 1: Ethical Dilemmas: Inpatient psychiatry comes with complicated ethical issues, including involuntary treatment, safeguarding and capacity assessments. This makes studying hospitalized patients, often in relapse, prospectively or during admission difficult, and often impossible. Participants interviewed reported that obtaining consent is difficult as it connects to patients’ capacity details, which requires access to medical records.

Theme 2: Systematic Healthcare and Documentation Issues: Mental health services and protocols at BPH have been constantly changing, adapting to resources and demand. This is reflected in documentation; admissions had different forms and therefore, information is not homogeneous throughout the years. Further complicating the already tedious manual data collection from the paper-based system. Despite the growth and interest in Palestinian psychiatry academia, it still lacks awareness, education, institutional support, and funding.

Theme 3: Clinical Setting: Being the only standing inpatient psychiatric facility in Palestine, the hospital’s wards are frequently crowded, under-resourced, and understaffed. Participants reported that many medical and psychological investigations and interventions are not available, and data collection needs to go through delicate paths to not “stand in the way” of patient care. BPH is a stand-alone psychiatric facility. Meaning there is no integrated system that connects it to other medical wards or outpatient clinics. This limits studying cases or phenomena that overlap with other specialties, and restricts outcome assessment and continuity of care.

Theme 4: Sociopolitical issues: The volatility of politics in Palestine renders all aspects of life unpredictable: from roadblocks to safety concerns, and patients’ mistrust in existing institutions. Writing about Palestinian mental health demands navigating medical “neutrality” without compromising critical social determinants of health. Finally, social challenges, including stigma, socioeconomic disparities, accessibility issues and cultural beliefs and misconceptions, hold patients and families back from sharing their information with investigators.

**Conclusion:**

Several hurdles prevent the publication of inpatient psychiatric research in Palestine. Researchers must be culturally sensitive and prioritize patient care and safety in vulnerable populations. The role of systematic change remains pivotal.

